# *Mycobacterium chimaera* Pulmonary Disease in Cystic Fibrosis Patients, France, 2010–2017

**DOI:** 10.3201/eid2503.181590

**Published:** 2019-03

**Authors:** Romaric Larcher, Manon Lounnas, Yann Dumont, Anne-Laure Michon, Lucas Bonzon, Raphael Chiron, Christian Carriere, Kada Klouche, Sylvain Godreuil

**Affiliations:** University of Montpellier, Montpellier, France (R. Larcher, M. Lounnas, C. Carriere, K. Klouche, S. Godreuil);; Montpellier University Hospital, Montpellier (R. Larcher, Y. Dumont, A.-L. Michon, L. Bonzon, R. Chiron, C. Carriere, K. Klouche, S. Godreuil)

**Keywords:** Mycobacterium chimaera, bacteria, Mycobacterium avium complex, tuberculosis and other mycobacteria, nontuberculosis mycobacteria, NTM, respiratory infections, cystic fibrosis, pulmonary disease, forced expiratory volume, forced vital capacity, France

## Abstract

We report *Mycobacterium chimaera* pulmonary disease in 4 patients given a diagnosis of cystic fibrosis in a university hospital in Montpellier, France. All patients had *M. chimaera*–positive expectorated sputum specimens, clinical symptoms of pulmonary exacerbation, or a decrease in spirometry test results that improved after specific treatment.

*Mycobacterium chimaera* is a member of the *Mycobacterium avium* complex, which was elevated to species rank in 2004. *M. chimaera* was reported by Tortoli et al. (*1*) as a cause of human lung disease but has been widely known as the bacteria responsible for an outbreak of endocarditis and disseminated infection after cardiac surgery in 2013 (*2*).

Although virulence and pathogenicity of *M. chimaera* in lung disease are currently debated, several cases of *M. chimaera* lung infections have been reported in settings of chronic obstructive pulmonary disease, malignancy, or immunosuppression (*3*–*5*). We found 1 case of *M. chimaera* infection in a patient with cystic fibrosis (*6*). Other nontuberculous mycobacteria (NTM), especially *M. abscessus* and *M. intracellulare*, are well-known pathogens in such a setting (*7*). We report *M. chimaera* pulmonary disease in 4 patients with cystic fibrosis.

After reviewing data for 248 patients who were examined at the Cystic Fibrosis Center of Montpellier, France, during 2010–2017, we observed that 24 (9.7%) of 248 patients had >1 respiratory smear sample positive for NTM; for 4 (16.7%) of 24, the sample was positive for *M. chimaera*. The 4 case-patients were Caucasian, age 8–21 years, and who had a newborn diagnosis of cystic fibrosis, preexisting respiratory impairment, and digestive malabsorption. The association of an increased cough and sputum production, breathlessness, and fatigue with a reduction in forced expiratory volume in 1 s (FEV1) or forced vital capacity (FVC) was diagnosed as pulmonary exacerbation (*7*) for all patients (Figure). Diagnosis was confirmed by computed tomography by the presence of bronchiectasis and nodules (tree-in-bud pattern) for case-patients 1 and 3.

**Figure:**
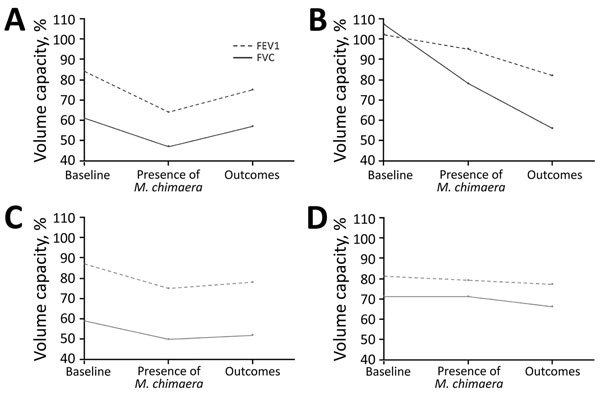
Evolution of lung function for 4 cystic fibrosis patients with *Mycobacterium chimaera* pulmonary disease, France, 2010–2017. A) Case-patient 1, B) case-patient 2, C) case-patient 3, D) case-patient 4. Case-patients 1 and 3 were given specific treatment for *M. chimaera* disease for 3 months; case-patient 2 was not given specific treatment; case-patient 4 was given only partial treatment. FEV1, forced expiratory volume in 1 s; FVC, forced vital capacity.

Respiratory specimens collected every 3–6 months for 1 year were digested and decontaminated by using the sodium dodecyl sulfate–NaOH method and then centrifuged using fluorescence microscopy. Sputum samples from all patients were negative for acid-fast bacilli. Samples were cultured on solid and liquid media (BACTEC MGIT 960 System; Becton Dickinson Diagnostic Systems, https://www.bd.com) which identified, after 11–41 days, mycobacteria from >2 separate sputum samples.

We performed species identification by using a commercial kit (GenoType NTM-DR assay; Hain Lifescience, https://www.hain-lifescience.de) and identified isolates as *M. chimaera*. We also performed molecular identification of isolates as *M. chimaera* as described (*1*,*8*). All isolates were susceptible to macrolides and aminoglycosides. We also isolated several other microorganisms: *Pseudomonas aeruginosa* and methicillin-resistant *Staphylococcus aureus* from case-patient 1; *Haemophilus influenzae* from case-patient 2; *M. avium* and *Stenotrophomonas maltophila* from case-patient 3; and *M. abscessus*, methicillin-resistant *S. epidermidis*, and *P. aeruginosa* from case-patient 4.

Azithromycin, rifampin, and ethambutol (in combination) and ceftazidime, tobramycin, and inhaled colistin were given to case-patient 1. No antimicrobial drugs were given to case-patient 2. Azithromycin and rifampin (in combination) and inhaled colistin were given to case-patient 3. Clarithromycin and linezolid were given to case-patient 4. All 4 case-patients required physiotherapy.

All case-patients were followed up for >1 year after the first positive smears for *M. chimaera* were obtained. We found a substantial reduction in symptoms of pulmonary exacerbations and sterilization of sputum specimens for patients given macrolides and rifampin with or without aminoglycosides after 3 months, as well as improvement in FEV1 and FVC after 6 months. In contrast, patients not given treatment (case-patient 2) or given only partial treatment with an anti-NTM antimicrobial drug regimen (case-patient 4) showed a decrease in FEV1 and FVC after 6 months (Figure) and slight recovery or no change after 1 year.

Therefore, we hypothesized that *M. chimaera* showed virulence and pathogenicity in our patients because of their clinical picture and evolution. We are aware that the diagnosis of NTM diseases according to American Thoracic Society criteria (*9*) might not be made with complete certainty because definitive exclusion of other diagnoses was often difficult for cystic fibrosis patients. Several confounding factors, such as co-infection with conventional pathogens, were observed, which could explain the observed favorable outcome. However, specific treatment against NTM improved outcome, which strengthens our presumption of the potential pathogenic role of *M. chimaera* in lung disease of patients with cystic fibrosis.

Cystic fibrosis transmembrane conductance regulator disorder results in mucus retention and bronchiectasis that favor repeated respiratory tract infection, including NTM diseases (*7*). In these circumstances, cystic fibrosis might promote *M. chimaera* infection in a similar manner to that in patients with chronic obstructive pulmonary disease. The lack of improvement of respiratory function for case-patient 4, who had been given treatment for infection with other pathogens, but only partially for *M. chimaera*, supports our hypothesis. However, whether *M. chimaera* is only a surrogate of respiratory impairment without any virulence or a real pathogenic microorganism remains unknown. In conclusion, *M. chimaera* lung disease should prompt physicians to consider this bacteria as an emergent pathogen in cystic fibrosis patients.
